# Promoting parkrun to patients using the general practice website: a qualitative exploration of ‘parkrun practice’ websites

**DOI:** 10.3399/BJGPO.2020.110

**Published:** 2021-03-10

**Authors:** Joanna Fleming, Rebecca Mensah

**Affiliations:** 1 Warwick Medical School, University of Warwick, Coventry, UK

**Keywords:** qualitative research, health promotion, information technology, primary health care

## Abstract

**Background:**

The parkrun practice initiative is a widely accessible and low-cost approach in primary care, which promotes patient and staff health and wellbeing through increased physical activity and volunteering opportunities. The parkrun practice Toolkit offers guidance to practices on how to promote parkrun. One recommendation is to include a parkrun page on the practice website, or a link to the parkrun website. How practices are presenting this information is unknown.

**Aim:**

To explore how parkrun practices are using their websites to promote parkrun, including the content and format of information presented and variety of promotion methods used, in order to provide recommendations for inclusion in the Toolkit.

**Design & setting:**

Qualitative content analysis of a sample of parkrun practice websites.

**Method:**

The websites of 114 practices that reported using their websites for parkrun promotion were systematically searched and the content analysed.

**Results:**

Five main types of content were identified, namely: what parkrun or parkrun practice is; activities and events; addressing patients’ concerns; benefits of parkrun; and practical information. While there were similarities in the information provided, there were variations in presentation. Websites ranged from being extensive and highly informative, to minimal amounts of text or solely images.

**Conclusion:**

Findings highlight the variability that currently exists across parkrun practice websites. Including a link on the homepage would assist patients to find information on parkrun and clarify the information that can be found. Suggestions are made on the type of information and how it could be presented to be further developed for inclusion in the Toolkit.

## How this fits in

The internet has become a key tool for obtaining health information by the public and while general practice websites in the UK offer a wide range of information, they may not fully exploit the potential for patient communication. The parkrun practice initiative encourages practices to link with their local parkrun events. Practices can use the parkrun practice for guidance on the types of activities that the practice can undertake, although none are mandatory and creativity is encouraged. The extent to which parkrun practices are using their practice websites is unknown and so this study explored how registered parkrun practices are using their websites to promote parkrun and, in doing so, identified implications for practice.

## Introduction

The parkrun practice initiative, launched by parkrun UK and the Royal College of General Practitioners (RCGP) in June 2018, is a widely accessible and low-cost approach in primary care to promoting patient and staff health and wellbeing through increased physical activity and volunteering opportunities.^1^ It encourages practices to link with local parkrun(s)^1,2^ and, to date, almost 1500 practices have registered. The signposting of patients to non-medical, community, and social activities, known as ‘social prescribing’ is becoming more common as a way to help patients manage and prevent illness, thereby improving their health and wellbeing.^3^ The parkrun practice initiative is one way in which a voluntary community organisation is being embedded into the wider primary care context. Practices can use the parkrun practice Toolkit,^1^ which offers guidance on activities; for example, displaying parkrun information on television screens, or sharing flyers with patients or colleagues. One recommendation is to include a parkrun page on the practice website, or a link to the parkrun website. How practices are presenting this information is unknown.

The internet is a key tool for the public to obtain health information.^4^ While parkrun practices are not delivering an internet intervention,^5^ the website offers a medium for patients to learn about parkrun, its benefits, and how to become involved. Practice websites offer a wide range of information, and while technically satisfactory, they often do not fully exploit the potential for electronic doctor–patient communication,^6^ and can be incomplete or lack coherence and strategy.^7^ As such, dissemination of public information is not always optimised.

The aim of this study was to explore how parkrun practices use their websites to promote parkrun by: 1) examining the content and format of the information presented; 2) examining the variety of promotion methods used; and 3) providing suggestions for how practices could use their website to promote parkrun.

## Method

### Design

A qualitative examination took place of a sample of practices that completed an online survey as part of a larger study about being a parkrun practice. A detailed explanation of the survey and sampling is published elsewhere.^8^ In the current study practices were asked the following question: 'Which activities suggested in the parkrun practice Toolkit have you adopted?' One of the options available was: 'Include a parkrun page on the practice website, or a link to the parkrun website.' Those who responsed 'undertaken' were included.

### Sample

An online survey was sent to all 780 parkrun practices in the UK,^8^ and 306 (39.2%) completed it. Of these, 114 (37.3%) reported including a parkrun page on their website, or link to the parkrun website, and were included in the current study.

### Data collection

Each website was systematically searched to identify parkrun content (October 2019). A small sample was initially searched with information location recorded; for example, homepage, news page. Many practices used a similar format, which provided a systematic approach to searching each one. Where present, pages were searched in the following order: homepage, news, newsletter, practice information, patient information, health advice, self-help, physical activity and fitness, social prescribing, services, and events. If no parkrun content was found, the search tool was used.

Screenshots of all parkrun content were saved. A data extraction proforma was used to collect descriptive data (for example, information location, and ease of finding). Ease of finding was determined by the number of ‘clicks’ needed to take the website user from the homepage to the specific parkrun page (Supplementary Appendix S1).

### Analysis

A content analysis was used to analyse the screenshots uploaded to NVivo (version 12). Content analysis explored and analysed the correlation between texts and possible themes or concepts.^9^ The analysis included the following components: 1) reading and familiarising with the data; 2) types of content noted and codes created; and 3) coding of data, primarily by one researcher with subsets of data coded by a second researcher to ensure accuracy.

Where discrepancies were present in coding, the two researchers discussed these to reach an agreement. All discrepancies were resolved without the need for a third researcher.

One researcher conducted this primary analysis, with a second involved in subsequent analytical discussions to describe the data within the context of the study aim.

## Results

One hundred and fourteen practices said they had included a parkrun page on the practice website, or a link to the parkrun website. Practice characteristics are shown in Table 1.

**Table 1.** Practice characteristics (*N* = 114)

**Characteristic**	**Value**	***n* (%**)
**Number of patients registered at practice**	<4000	2 (1.8)
	4000–8000	26 (22.8)
	12 000–16 000	28 (24.6)
	16 000–20 000	16 (14.0)
	>20 000	8 (7.0)
**Number of parkruns linked to**	0	1 (0.9)
	1	103 (90.4)
	2	3 (2.6)
	3	2 (1.8)
	4	0 (0.0)
	≥5	3 (2.6)
	Not stated	2 (1.8)
**Closest parkrun is within patient catchment area**	Yes	78 (68.4)
	No	34 (29.8)
	Not stated	2 (1.8)
**Practice had involvement with parkrun before initiative**	Yes, a lot	2 (1.8)
	Yes, a little	50 (43.9)
	None at all	61 (53.5)
	Don’t know	1 (0.9)

Almost half (47.4%) had between 4000–16 000 registered patients. Most (90.4%) were linked with one parkrun, but three (2.6%) were linked with ≥5. In two-thirds (68.4%), the closest linked parkrun was within the practice’s catchment area. Just over half (53.5%) had no involvement with parkrun before the initiative, and 43.9% a little prior involvement. Of the 114 practices reporting using their website, 79 (69.3%) were currently doing so.

### Website characteristics

**Figure 1.**
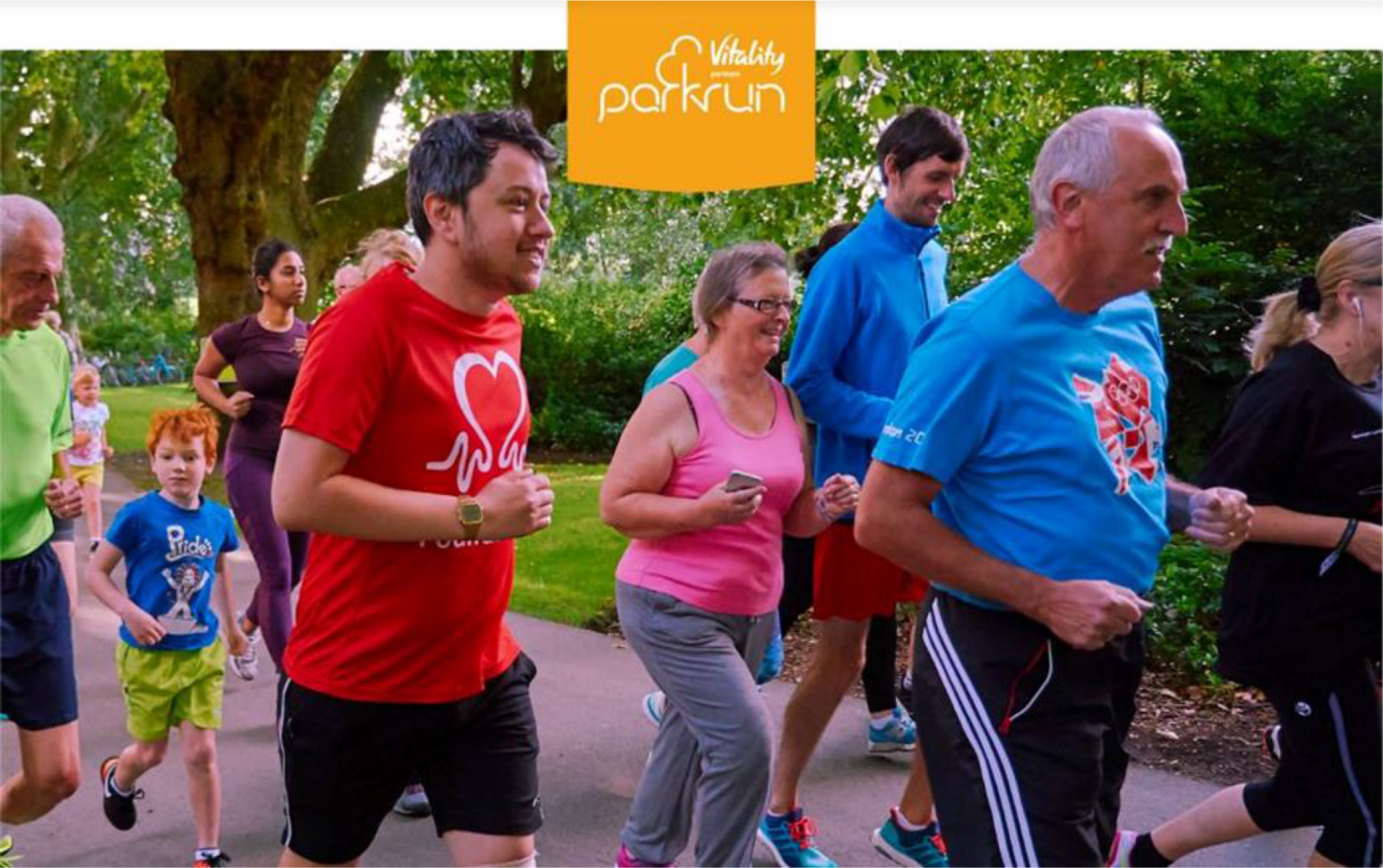
Most commonly used image.

Data were recorded about type, location, and frequency of information presented on practice websites (Table 2). Over half (*n* = 43/79; 54.4%) provided parkrun information, linked to parkrun information, or included the parkrun logo on their homepage, while for some, parkrun information was located in dated news bulletins, or downloadable newsletters. Over a quarter used these to provide current news (*n* = 23/79; 29.1%). While easy to find at the time of publication, this was more difficult once archived. Just under a quarter (*n*= 18/79; 22.8%) had information in other areas, including social prescribing pages, practice information, and self-help pages. To find information here, websites were manually searched. Text alone was the most common format, with those not using text commonly using the official parkrun logo or flyer. Many practices used the same text and bullet-point layout made available in the Toolkit.^1^ A commonly used image, available in the Toolkit as an official flyer,^1^ involved a group of people of mixed age and sex participating together (Figure 1). Other images included mothers running with buggies and people running with dogs on leads. Some practices used social media (for example, Twitter and/or Facebook) to promote parkrun or to post images and/or YouTube videos from recent events or notices.

**Table 2.** Type, location, and frequency of information presented (*N* = 79)

**Type of information**	***n* (%)**
**Content and location**	
parkrun information on homepage	18 (22.8)
Link to parkrun information on homepage	19 (24.1)
parkrun logo on homepage	6 (7.6)
parkrun information on news page	19 (24.1)
parkrun information in newsletter	4 (5.1)
parkrun information in other areas of website	18 (22.8)
Use of official parkrun literature (for example, leaflets)	42 (53.2)
Links to other health initiatives	19 (24.1)
Use of social media(for example, Facebook, YouTube, news articles)	20 (25.3)

Five types of content were identified:

- what parkrun or parkrun practice is;
- parkrun practice activities and events;
- addressing patients’ concerns about participating in parkrun;
- benefits of parkrun (health and other); and
- practical information.

Table 3 shows how often this information was presented across the 79 parkrun practices. The most common were ‘what parkrun or parkrun practice is’ (*n* = 65; 82.3%), and practical information (*n* = 60; 75.9%). Reporting activities and events was least common with 28 (35.4%) doing so.

**Table 3.** Presentation of types of information (*N* = 79)

**Type of information**	**parkrun practices presenting this information, *n* (%**)
What parkrun or parkrun practice is	65 (82.3)
parkrun activities and events	28 (35.4)
Addressing patients’ concerns about participating in parkrun	54 (68.4)
Health benefits of parkrun	40 (50.6)
Other (non-health) benefits of parkrun	42 (53.2)
Practical information	60 (75.9)

#### What parkrun or parkrun practice is

Details about what parkrun is were similar across websites with the same terminology used, namely: *‘Free, weekly, timed 5 km events taking place every Saturday morning.’* Some stated solely that they were a ‘parkrun practice’, whereas others explained more about the partnership and its aims and objectives.

#### parkrun practice activities and events

Use of official parkrun literature provided some consistency with parkrun activities and events.

Some practices provided information about activities they had done or were planning, such as ‘parkrun volunteer takeovers’ where staff take on all the volunteering roles at their linked parkrun on a specific date (for example, marshalling, time-keeping, barcode-scanning). This was often accompanied by photos of staff wearing volunteer jackets. Some practices had completed a parkrun together and showed photos of staff at the event. There was sometimes mention of recurring occasions when staff participated in parkrun; for example, *‘Our practice meets on the first Saturday of every month,’* or future plans to complete a parkrun takeover; for example, *‘On the 10^th^ November we will be staffing the [park name] event. Why not come and join us?’*

Sometimes parkrun information was located alongside information about other physical activities, including the cancer support group 5k Your Way,^10^ walking groups, and dancing groups for people aged ≥65 years.

#### Addressing patients’ concerns about participating in parkrun

Concerns addressed included ability, speed, fears, and support. parkrun was described by some as not being a race and patients did not need to be experienced; for example, *‘We were all really pleased to learn that parkrun is NOT A RACE.’* There was reassurance about suitability for all abilities; for example, ‘[parkrun is] *open to everyone, including those who are inactive or have health conditions or disability.’* It was made clear that speed did not matter and patients were free to go at a pace that suits them. Images to show different groups of people running together, with a sense of enjoyment were frequently used. The most commonly used image (Figure 1) is the parkrun flyer available in the Toolkit.^1^

#### Benefits of parkrun (health and other)

Described benefits included both health and other benefits. Health benefits included improved fitness, physical and mental health, and social wellbeing. parkrun was commonly described as *‘a great way to cope with health problems’*, or to lessen the impact of chronic health conditions. Some gave specific examples of where parkrun could be beneficial, such as diabetes management, weight loss, and mental health; for example, depression, anxiety, and low self-esteem. A commonly described benefit was social wellbeing. Many referenced the welcoming and supportive community of parkrun, which could create a good opportunity to socialise and make friends; for example, post-event socialising, where people could *‘maybe enjoy a nice coffee and chat at the end’*.

#### Practical information

Details on how to prepare and what to expect at parkrun were present on some websites. Some gave direct links to the parkrun registration page or homepage. Details of parkrun location were often varied; some specifically named the venue, while others provided postcodes and maps detailing meeting points. Little information was given about running route, terrain, and number of laps; however, if a link to the event website was included, this was detailed here. Some practices provided details of people they could contact for more information, ranging from *‘any member of the practice team’* to named staff members.

## Discussion

### Summary

This study identifies the content and format of information provided by parkrun practice websites in the promotion of parkrun to their patients. While there were some similarities in the text and images used, there were variations in layout and presentation. Websites ranged from being extensive and highly informative, to having minimal amounts of text or images. Findings demonstrate the variability that currently exists across websites and provides suggestions for information to be included. With refinement, this information could be an addition to the Toolkit. Findings highlight the importance of practices becoming further embedded within their local communities and signposting patients to existing community services, as well as the importance of the accessibility of updated and timely information on practice websites, ease of access, and user-friendliness.

### Strengths and limitations

To the authors' knowledge, this is the first study to investigate information provided on parkrun practice websites where analysis of the content has enabled a detailed investigation. The data are based on practices responding to a survey sent in April 2019, less than a year since the initiative started, and so some had not been part of the initiative for long. Half (*n* = 155/306; 50.7%) reported *‘intending to use their website to promote parkrun’*. It is likely that, over 12 months on, there will be more practices utilising their websites. Furthermore, those already using them may have increased or improved the information being provided, although it is likely that the issues of variability still remain.

### Comparison with existing literature

The GP Patient Survey is an independent survey run on behalf of NHS England. It is sent out to over 2 million people across the UK each year to assess how people feel about their practice. In 2019, it showed just under two-fifths of patients (38.2%) tried to use their practice website to access information or services; an increase of 2.7% compared with 2018.^11^ Of patients who tried, 77% found it easy to use.^11^ Previous research has found webpages that receive high scores of pleasure correlate with low and medium levels of complexity; simple and moderately complex websites were liked more.^12^ The homepage is the first aspect visitors will see. In the current study, practices where parkrun information or link was located on the homepage were easy to navigate, as the information did not need to be located on other pages. Having information clearly visible on the homepage may allow patients to chance upon it and take interest. However, parkrun is likely to be competing with other important aspects, such as information regarding registering and appointments. Therefore, ways in which parkrun can be incorporated, for example, a clearly visible link to further information, may help this.

A number of factors are important in contributing to attendance and sustained involvement in parkrun, including event accessibility, opportunities for social interaction, the outdoor environment, and volunteering.^13^ The majority of websites portrayed parkrun as an event ‘open to everyone’ where ‘all are welcome’, which aligns with findings of ‘reciprocity’ and ‘freedom’.^13^ Two-thirds of practices had a parkrun within the practice catchment area. That said, recent findings have shown that improving geographical access by creating new parkrun events is unlikely to substantially increase the number of participants from lower socioeconomic groups.^14^ Having a nearby parkrun and available website information is only a small part of this initiative.

Addressing concerns and challenging perceptions of parkrun — such as speed, fear, and ability — was prominent across websites. The parkrun PROVE project, which aims to increase engagement in parkrun by those living with long-term health conditions, has shown success to be dependent on parkrun being accepted as an activity appropriate for all abilities and breaking down any misconceptions that could act as a deterrent.^15^ The findings of the present study show many practices addressing these issues. However, further information specific to patient groups, such as those with long-term health conditions, may be useful. Websites could further highlight the benefits of parkrun to specific patient populations, whereby patients who have benefitted from engaging in parkrun could become local practice champions. The champions could share their success stories and empower other patients with similar conditions, using the website and other mediums such as social media.

### Implications for research and practice

Using the practice website is just one activity suggested in the Toolkit. The initiative is low maintenance, with practices left to do what suits them best. Further guidance on how a webpage might look and what information to include could be beneficial. Where practices are already pushed for time to carry out activities,^8^ and have heavy practice staff workloads,^16^ making website use as easy as possible may increase adoption.

**Figure 2.**
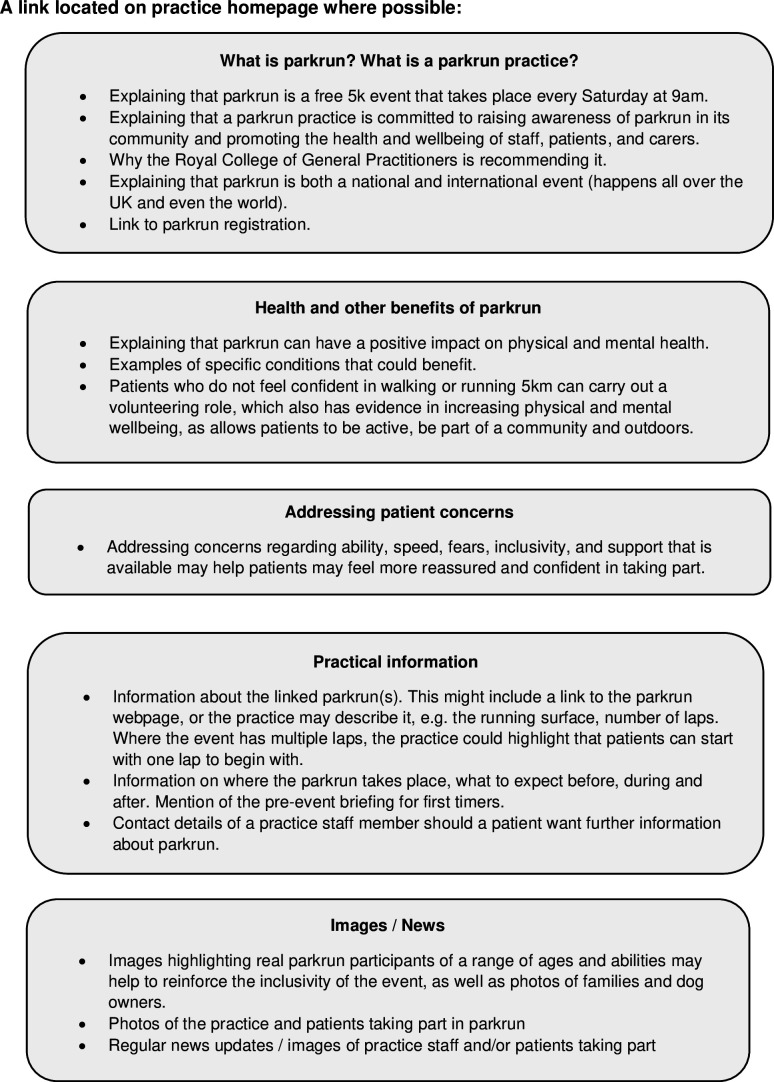
Parkrun practice information.

Collating evidence from practices that currently use their websites to promote parkrun has identified a possible template that could be added to the Toolkit and tested (Figure 2).

It is unknown how many people visit practice websites for parkrun information or generally. Further research, including hit rates for specific pages, alongside research with patients assessing the ease of accessing information, would be useful in understanding their use and how to make more targeted evidence-based recommendations. Used in combination with other suggested Toolkit activities, an improved website may help increase patient participation.

Of the 114 practices that reported using their website, only 79 (69.3%) were currently doing so. This could be owing to, for example, not locating the information, or information being removed, or not yet added. Future research could include learning about the challenges associated with keeping website information current and how this could be overcome. While this study did not look specifically at social media, some had links to these. Further exploration on how practices could use social media to promote parkrun and how this might influence patients’ actions in conjunction with website use would be beneficial.
